# Response to: Self-Directed Interventions to Promote Weight Loss: A Systematic Review of Reviews

**DOI:** 10.2196/jmir.3476

**Published:** 2014-07-24

**Authors:** Melinda J Hutchesson, Philip J Morgan, Penelope McCoy, Clare E Collins

**Affiliations:** ^1^School of Health SciencesFaculty of Health and MedicineUniversity of NewcastleCallaghanAustralia; ^2^Priority Research Centre in Physical Activity and NutritionUniversity of NewcastleCallaghanAustralia; ^3^School of EducationFaculty of Education and ArtsUniversity of NewcastleCallaghanAustralia; ^4^SP Health Pty LtdNorth SydneyAustralia

In the February issue of the *Journal of Medical Internet Research*, Tang et al reported a systematic review of reviews examining the effectiveness of self-directed interventions to promote weight loss [1]. They reviewed 20 systematic reviews which incorporated 99 primary studies relevant to their review question. They concluded that self-directed interventions promote weight loss both independently and when provided as an adjunct to personal contact interventions.

We agree that the evidence presented in this systematic review of reviews provides some support for the use of self-directed interventions for weight loss; however we believe the data provided does not specifically support the use of self-directed interventions alone and when used in combination with other delivery modes. We wish to point out that this is because the majority of reviews included do not have inclusion criteria or present results in a way that considers whether the intervention was delivered solely in a self-directed format or in combination with other delivery modes (eg, face-to-face). In most of the reviews, these two types of self-directed interventions are grouped together when results are presented in a narrative summary or meta-analysis. We believe that as part of their recommendation for a comprehensive review of primary evaluations, the authors should have recommended that future systematic reviews of self-directed interventions should consider results separately for interventions that are delivered *solely* using self-directed modes, and those that use self-directed modes *in combination* with other delivery modes. Furthermore, we believe that authors should be encouraged to describe the components of self-directed modes in sufficient detail to enable such a classification.

We are also concerned about the accuracy of the methodological quality review of Tang et al. For example, the review incorrectly states that Neve et al [2] “did not use quality assessment”. However, the methods section of this review reports that “included studies were assessed by two independent reviewers for methodological validity using a standardized critical appraisal instrument from the JBI Meta-Analysis of Statistics Assessment and Review Instrument” (p 307) and the tool is provided as an Appendix to the paper [2]. Furthermore, the results include a description of the critical appraisal findings (subheading “Quality of Included Studies” p 310)[2]. As the results of the review of methodological quality are only presented as an overall score, and not by the individual criteria, it is difficult to determine how this influences the results of the quality evaluation within the review by Tang et al [1].

We agree with the authors that meta-analyses of self-directed weight loss interventions should be undertaken to help identify what intervention techniques and delivery formats are most effective. However, as an author of one of the included systematic reviews, we are aware that the absence of such meta-analyses from the included reviews is likely due to inadequate descriptions of intervention techniques and delivery formats currently being published. Therefore, we believe a more important recommendation is that researchers who are currently evaluating self-directed interventions should provide more robust descriptions of their interventions, as recommended by the CONSORT-EHEALTH statement [3].  Furthermore, this may be achieved by specifically publishing study protocols (eg, [4,5]), or descriptions of the intervention development methods (eg, [6]). 
